# University-based initiatives towards better access to oral health care for rural and remote populations: A scoping review

**DOI:** 10.1371/journal.pone.0217658

**Published:** 2019-05-31

**Authors:** Richa Shrivastava, Frances Power, Farzeen Tanwir, Jocelyne Feine, Elham Emami

**Affiliations:** 1 Faculty of Dentistry, Université de Montréal, Montreal, Quebec, Canada; 2 Faculty of Dentistry, McGill University, Montreal, Quebec, Canada; University of Queensland, AUSTRALIA

## Abstract

This scoping review maps a wide array of literature to identify academic programs that have been developed to enhance oral health care for rural and remote populations and to provide an overview of their outcomes. Arksey and O’Malley’s 5-stage scoping review framework has steered this review. We conducted a literature search with defined eligibility criteria through electronic databases, websites of academic records, professional and rural oral health care organizations as well as grey literature spanning the time interval from the late 1960s to May 2017. The charted data was classified, analyzed and reported using a thematic approach. A total of 72 citations (67 publications and seven websites) were selected for the final review. The review identified 62 universities with program initiatives towards improving access to oral health care in rural and remote communities. These initiatives were classified into three categories: training and education of dental and allied health students and professionals, education and training of rural and remote community members and oral health care services. The programs were successful in terms of dental students’ positive perception about rural practice and their enhanced competencies, students’ increased adoption of rural practices, non-dental health care providers’ improved oral health knowledge and self-efficacy, rural oral health and oral health services’ improvement, as well as cost-effectiveness compared to other strategies. The results of our review suggest that these innovative programs were effective in improving access to oral health care in rural and remote regions and may serve as models for other academic institutions that have not yet implemented such programs.

## Introduction

Dental workforce shortages in rural and remote areas have been reported throughout the world [1–8]. Educational and socio-economic background, altruistic motivation, previous life experience, and exposure to rural and remote community activities have been shown to influence dental professionals’ decisions in their choice of practice location and willingness to work in a rural and remote area [9–11]. Shortages of dental professionals can lead to reduced accessibility to oral health services and poorer oral health status for rural dwellers than for urban populations [2, 7, 12–15]. It has been reported that people living in rural and remote areas have more unmet dental care needs, poorer oral health knowledge and practices and higher rates of dental caries [14, 16, 17].

The World Health Organization has proposed three strategies to improve access to health workers in rural and remote areas: education and regulatory interventions, monetary compensation and management, environment and social support [18]. A variety of strategies have been recommended to resolve disparities in access to oral health services: prevention and promotion through public health approaches, such as water fluoridation and school-based interventions; facilitating infrastructure and technologies through E-health; temporary services through fly in—fly out or mobile clinic services; financial incentives for the dental workforce in the form of scholarships; interdisciplinary approaches to integration of oral health within primary health care; and academic strategies such as rural training and selective recruitment [7, 16, 19, 20]. Educational institutions have developed strategies to overcome problems due to dental workforce shortages, such as the provision of rural training and outreach programs for dental students, oral health training for allied healthcare professionals and students and selective admission of rural applicants [7, 16]. The impact of academic initiatives on an increased rural dental workforce and the concomitant promotion of rural oral health status is less clear, thus emphasizing the need to conduct this comprehensive review.

Over the past decades, various knowledge synthesis methods, such as narrative, integrative, realist, scoping and systematic reviews have been introduced to foster evidence-informed health care [21]. In 2001, Mays, Roberts, and Popay stated that the objective of a scoping review is to rapidly map the fundamental concepts, primary sources and types of evidence on a topic that has not yet been comprehensively reviewed [22]. We mapped a large body of literature to identify rural and remote academic programs and to give an overview of their outcomes, regardless of the quality of the included studies [22].

## Materials and methods

The Arksey and O’Malley’s scoping review 5-stage framework has steered this review [23]. Accordingly, the scoping review included five steps, as detailed below:

### 1. Identifying the research question

One specific research question guided the selection of relevant literature for this scoping review: What are the academic programs and their outcomes that have been designed to enhance oral health care for rural and remote populations?

### 2. Identifying relevant studies and eligibility criteria

Pertinent publications that spanned the time interval between the late 1960s and June 2017 were reviewed. The authors searched for publications by using Ovid (MEDLINE and Embase) and PubMed electronic databases. The search strategy (Table 1), designed for the MEDLINE database search, was later adapted for other databases. The electronic search was completed by hand searching the list of references in the identified publications or relevant reviews. Data were also retrieved from the websites of pertinent universities, as well as relevant professional, rural and remote oral health organizations. We included publications written in English only, in which academic institution initiatives on rural oral health care were the focus of the publications. After title and abstract screening, articles were excluded which showed no focus on university-based initiatives on rural oral health. Some of the articles were also excluded after full-text review (30) which were focused on rural oral health initiatives but lacked any interventions. Although editorials, commentaries, and reviews were excluded, their references to the original studies were searched and included in our study.

**Table 1:** Medline search strategy.

#	Searches
1	Education, Professional
2	exp Schools, Dental/
3	exp Students, Dental/
4	Community Health Services/ or Community-Institutional Relations/ or "Delivery of Health Care"/ or Health Education/
5	exp Universities/
6	Clinical Competence/
7	exp Oral Health/ or exp Dental Health Services/ or exp Dental Care/
8	Dentists/
9	Dental Auxiliaries/
10	Dental Facilities/
11	Dentistry/ or Public Health Dentistry/ or Community Dentistry/ or Preventive Dentistry/ or Pediatric Dentistry/ or Dentistry, Operative/ or School Dentistry/ or Geriatric Dentistry/
12	exp Rural Health Services/ or Rural Population/ or exp Rural Health/
13	Medically Underserved Area/ or Health Services Accessibility/
14	Telemedicine/
15	1 or 2 or 3 or 4 or 5 or 6
16	7 or 8 or 9 or 10 or 11
17	12 or 13 or 14
18	15 and 16 and 17
19	limit 18 to (English)

### 3. Study selection

Two independent reviewers (RS, EE) screened the titles and abstracts of each citation and identified eligible articles for full review. Disagreements were discussed and resolved by consensus.

### 4. Charting the data

One reviewer (RS) charted all data obtained from the selected publications based on authors, years, country, type of publication, program description, program outcomes measures, and results. The other reviewer (EE) then randomly checked 10% of the extracted data to ensure accuracy. Any noted discrepancy was rectified by consensus.

### 5. Collating, summarizing, and reporting the results

The charted data were summarized and reported using descriptive a numerical summary and qualitative thematic analysis approach. Investigator triangulation was conducted by the scoping review team (RS, EE, FP, FT, JF) who reviewed the charts, results and outcome measures.

## Results

### Characteristics of the included publications

**Fig 1:**
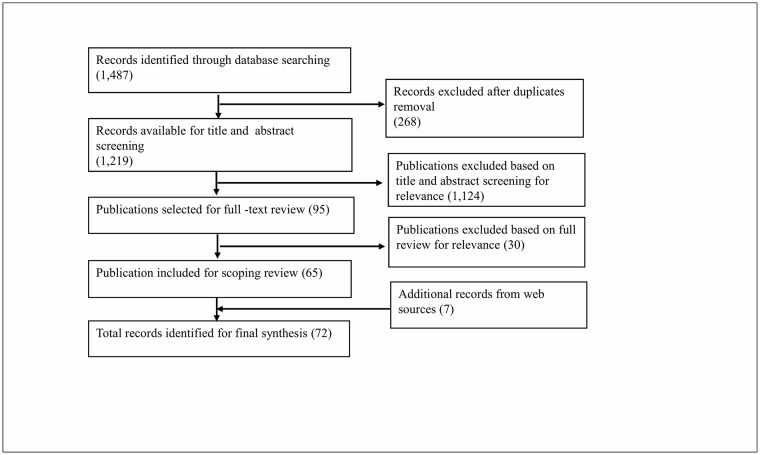
Flow diagram of search strategy.

Electronic and hand searches generated 1,487 records (Fig 1). After removal of duplicates, the title and abstract screening was conducted for 1,219 citations, out of which 95 articles were selected for full-text review. From these articles, 65 publications met the eligibility criteria for the scoping review. Additional information was found from 7 healthcare or educational organizations’ web records that were relevant to our scope of review. The inclusion of these records then generated a total of 72 records for final synthesis.

The scoping review identified a total of sixty-two universities taking initiatives towards improving access to oral health care in rural and remote communities. These publications were identified from 16 countries: USA, Canada, Australia, New Zealand, United Kingdom, Scotland, Malta, Brazil, Peru, India, China, South Africa, Nigeria, Uganda, Romania, and Bulgaria. Most of the included publications were from North America, Asia, and Australia and were published in the last decade.

## Program classification

Based on our scoping review results, we identified three categories of programs that have been implemented in various universities. The first category characterizes programs for the training and education of dental and allied health students and professionals [1, 3, 11, 24–67]; the second category describes programs for the education and training of rural and remote community members [68–73] and the third category represents programs on oral healthcare services in rural and remote areas [41, 42, 61–63, 68, 69, 73–92].

### Themes identified in these university-based rural oral health initiatives

All included programs were clustered into the following four themes identified as implementation platforms. These were the curriculum-based platform; joint programs with the public health sector, organizations and community platform, E-health platform, and mobile dentistry platform (Table 2). Some of the identified programs overlapped under these platforms due to their common objectives.

**Table 2:** Description of program platform themes based on program categories.

Platforms	Programs for training and education of dental and allied health students and professionals	Programs for education and training of rural and remote community members	Programs for oral health care service in rural and remote areas
Curriculum-based	Rural training and courses for dental students (mostly 4^th^, 5^th^ grade, and internships) Placement programs (1–10 weeks) [1], [3], [24], [25], [26], [27], [28], [29], [30], [31], [32], [33], [34], [35], [36], [37], [38], [39], [40], [41], [42], [43] Dental education courses [44], [45], [46], [47], [48], [41], [42], [49] Outreach programs [50], [51], [52] Postgraduate fellowship program [53] Rural, under-represented minority and low-income students to study and practice dentistry in rural areas [54], [55], [56], [57], [58] Rural training and courses, including rural placements for allied health professionals and students [43] (Aboriginal health workers [59], medical and nursing students [33], family medicine residents [11], [60], pediatrics residents [61], [62])	Children’s oral health education [68], [69], [70] Patient education [71] Training for school teachers [70]	Provision of comprehensive oral health services [68], [69], [74], [75], [76], [77] Improvement of quality of oral health services and meeting community’s oral health needs [61], [62], [78], [41], [42] Delivery of cost-effective services [79], [41], [42]
Joint programs with the public health sector, organizations and community	Training for health workers [63]	School teachers’ training [72] Children’s oral health education [72]	Oral health promotion and prevention [63], [80] School-based oral health education and services [81] Provision of culturally sensitive oral health care by Aboriginal communities recruiting its own dentist, dental assistant and health worker [82], [83]
E-health	Tele-dentistry training for allied dental workforce [64]		Video consultation with the specialist to discuss treatment options and prescriptions [84] Virtual dental home for risk assessment, preventive, and operative services and follow ups [85]
Mobile dentistry	Training of students in dentistry and allied dental professions through mobile dental outreach [65], [66], [67]	Patient education [73]	Oral examination and consultation, oral health services especially preventive and curative services, referral services [86], [73], [87], [88] Improvement of oral health status [89] and meeting population’s oral health [90] Cost analysis [91], [92]

1. **Curriculum-based platform**: This platform incorporated various programs under the first category, classification of training and educational programs for dental students and allied health care professionals and students [1, 3, 11, 24–62]. These programs included 1 to 10 weeks of rural placement training for dental students (mostly fourth and fifth years and internship level) and dental hygiene students; dental education courses; outreach programs; postgraduate fellowship programs; and programs to encourage rural students, under-represented minority and low-income students to study and practice dentistry. The platform for the second category of education and training programs for rural and remote community members included patient oral health education and rural school teachers’ training [68–71]. Lastly, the curriculum based platform for programs in the third category of oral health care services incorporated programs for providing and improving oral health care services and fulfilling the community’s oral health-related needs [41, 42, 61, 62, 68, 69, 74–79].
2. **Joint programs with the public health sector, organizations and community platform**: This platform for the first category of training and education programs for dental and allied healthcare professionals and students included training of health workers [63]. Aboriginal health workers were responsible for managing patient appointments and communications, as well as oral health promotion activities with the dentists [63]. For its second category of classification, this platform incorporated training of school teachers and the oral health education of children [72]. Finally, for the third category, this platform included programs for oral health promotion: school-based oral health education and services and culturally-sensitive oral health care programs with community-led recruitment of its dentist, dental assistant, and Aboriginal health worker [63, 80–83].
3. **E-health platform**: This platform offered teledentistry that facilitated the training of an allied dental workforce for the first category of classification [64]. No relevant article was found in relation to the second category of the classification.
  This platform for the third category of classification included programs focused on oral health services through video consultation with dental specialists and a virtual dental home concept (telehealth dental home) for risk assessment, preventive and operative services and follow-ups [84, 85].
4. **Mobile dentistry platform**: This platform offered programs for the training of students in dentistry and allied dental professions by providing them with experience in mobile dental outreach under the first category [65–67]. It included programs focused on patient education for the second category [73]. Finally, for the third category, it encompassed programs that provided oral examinations and consultation, as well as preventive, curative and referral oral health services that improved patients’ oral health status [91, 92].

### Evaluations of programs (Tables 3, 4, 5 and 6)

**Table 3:** Summary of published research articles identified in the scoping review (1969–2005).

**Author; Year/ Country**	**University/ Institution**	**Type of publications**	**Program description**	**Outcome variable/ Measurement instrument**	**Results**
Podshadley AG, et al.; 1969/ USA [41] Heise AL, et al., 1973/ USA [42]	University of Kentucky	Original research report	“Community Clinical Laboratory” as 6 hours’ course for final year dental students providing comprehensive dental care for rural children with mobile dental units	Effect on oral health status, cost-effectiveness, and students’ competencies/Oral examination, cost analysis, questionnaire	Improved children’s oral health status Students had positive and new learning experience Cost-effective compared to private practice
Kurtzman C, et al.; 1974 [88]	University of California at Los Angeles and University of Southern California	Original research report	Mobile Dental Project for agriculture workers’ children in rural southern California by dental and dental hygiene students from all classes	Effect on oral health services/Descriptive measurement	Improved oral health care services Successful met their oral health-related needs
McMillan WB, et al.; 1975 [35]	University of Minnesota	Original research	Summer rural dental externship program for third-year dental students	Students’ competencies; dentists’ and students’ satisfaction/Pre-and post-questionnaires	Dental students’ positive attitude and preference for rural practice Most aspects for satisfaction were rated above average and excellent by dentists and students
Bentley JM, et al.; 1983/ USA [68]	University of Pennsylvania	Original research (experimental study)	Rural Dental Health Program for rural children randomly assigned to school-based practice group and private practitioners’ group that was further divided into improved dental health program and regular health program.	Comparing utilization of services by children over three years/Descriptive measurement	Increased dental service utilization by children assigned to school-based practice group offering improved dental health program in contrast to other groups
Feldman CA, et al.; 1988/ USA [69]	University of Pennsylvania	Follow up study	Follow up of the Rural Dental Health Program by Bentley JM, et al.; 1983 [68], evaluated seven years after the funding ended	Long-term evaluation after seven years/Health and oral health-related indices	Children assigned to community group utilized more professional services and showed more dental knowledge compared to another group with reduced service utilization when project ended
Shreve WB, et al., 1989/ USA [44]	University of Florida (contract basis with Lafayette-Suwannee Rural Health Corporation, Inc.)	Original research	Extramural 2-weeks dental education program for dental students	Impact on education, research and services/In-house and external evaluation and surveys	Met curricular guidelines and educational objectives of the American Association of Dental Schools Offered good educational experience for dental students Provided comprehensive oral services for rural population and improved their oral health knowledge
**Author; Year/ Country**	**University/ Institution**	**Type of publications**	**Program description**	**Outcome variable/ Measurement instrument**	**Results**
Burger AD, et al.; 1997/ USA [65]	Youngstown State University	Original research	Dental Disease Prevention and Early Intervention Program to train dental hygiene students and provide essential services to rural population: senior dental hygiene students worked in pairs with supervision of dentist using a mobile dental unit	Change in oral health service provision and students’ attitude and experience/Descriptive measurement and survey questionnaire	Mostly preventive and simple restorative dental services were provided Students reported positive experience Improved competencies and social sensitization
Pacza T, et al.; 2001/ Australia [59]	University of Western Australia	Original research (pilot program)	Oral health training program for rural and remote Aboriginal health workers to implement a culturally sensitive preventive oral health care delivery program	Attitude of health workers/Questionnaires and focus group discussions	Gained pleasant experience and willingness to administer the long-run preventive program
Kaakko T, et al.; 2002/ USA [74]	University of Washington	Original research (randomized clinical trial)	Access to Baby and Child Dentistry (ABCD) program involving Medicaid- enrolled children in rural Stevens County compared with children who had regular benefits	Assessment of oral health status, utilization rate and expenditure for Medicaid-enrolled children compared to children with regular benefits/Oral examination and descriptive measurement	Significantly improved oral health and utilization rates among ABCD children No cost difference between 2 groups
Richards L, et al.; 2002/ Australia [79]	University of Adelaide	Clinical report	Final year dental students posted in rural public dental service clinics at Whyalla and Port Augusta	Cost and benefits analysis/Evaluation of the effect on the waiting list, cost per patient during the course and marginal cost	Reduced waiting list and increased number of patients Services provided by students or private providers were more valued, and total cost of the treatment provided by students was found to be greater than public-sector dentists but less than private providers
Mouradian WE, et al.; 2003 [11]	University of Washington	Original research	Interdisciplinary Children’s Oral Health Promotion Project at University affiliated Family Practice Residency Network to train family medicine residents	Residents competencies and their ratings for instructors/Questionnaires	Improved family medicine residents’ competencies Residents evaluated course quality and instructors’ skills as ‘very good’
Gonsalves WC, et al., 2004/ USA [60]	University of Kentucky	Original research	Physicians’ oral health education for family medicine residents on children’s oral health screening, risk assessment, and counseling	Competencies of residents/Pre-test and post-test survey questionnaires	Improved knowledge, attitude, self- efficacy, and basic oral health care skills of family medicine residents
**Author; Year/ Country**	**University/ Institution**	**Type of publications**	**Program description**	**Outcome variable/ Measurement instrument**	**Results**
Woronuk JI, et al.; 2004/ Canada [37]	University of Alberta	Original research	Satellite dental program for third and final year dental students	Students’ evaluation by supervising dentists based on four categories: knowledge of preparatory phase, delivery of procedures, patient management and maintenance of infection control standards/Grading scales (1–4 for first three categories and 1 or 2 for fourth category)	Most students fulfilled their treatment objectives and were highly motivated with improved clinical competencies
Elkind A, et al.; 2005/ UK [51]	University of Manchester	Original research (pilot project)	Pilot outreach program for final year dental students in restorative dentistry and clinical sessions at the dental hospital	Students’ competencies/ Questionnaire	Outreach and clinical sessions benefitted each other: Outreach sessions offered treatment planning, emergency care, improved clinical experiences and time management skills whereas clinical sessions offered specialized teaching and improved their clinical competencies
Parker EJ, et al.; 2005/ Australia [82]	University of Adelaide	Preliminary project report	The culturally-sensitive oral health program for the Aboriginal community in Port Augusta—first phase in partnership (with Pika Wiya Health Service, South Australian center for rural and remote health and South Australian Dental service)	Community acceptance and change in the demand for services/Descriptive measurement	Accepted by the community with immediate demand for services Successful implementation of dental care service by recruiting its own dentist, dental assistant, and Aboriginal health worker

**Table 4:** Summary of published research articles identified in the scoping review (2006–2010).

**Author; Year/ Country**	**University/ Institution**	**Type of publications**	**Program description**	**Outcome variable/ Measurement instrument**	**Results**
Bernabe E, et al.; 2006/ Peru [47]	Universidad Peruana Cayetano Heredia	Original research	Dental Public Health Teaching-learning experiences of dental students in low income communities	Students’ competencies and experience/ Qualitative interviews	Good clinical competencies and enhanced sense of social responsibility among students
Harrison RL, et al.; 2006/ Canada [61]	University of British Columbia	Original research report	Brighter Smiles program trained pediatric residents in a remote First Nations community including brush-ins, fluoride application, oral presentations, and regular visits by pediatric residents	Improvement in the oral health of children in a remote First Nations community/Oral examination	Improved oral health of children Increased proportion of preventive services and significantly reduced the time needed for extraction of primary teeth by therapists
**Author; Year/ Country**	**University/ Institution**	**Type of publications**	**Program description**	**Outcome variable/ Measurement instrument**	**Results**
Bazen JJ, et al.; 2007/ Australia [3]	University of Western Australia	Original research	Rural, remote, and Aboriginal pre-graduation placements for dental students under the supervision of dentists	Students’ competencies and supervisors’ attitude/ Closed and open-ended questionnaires	Positive perception among students and more students preferred rural dental practice Supervising dentist graded students’ clinical competencies and their relationships with patients and local staff as ‘very good’ < 1/3^rd^ of the students believed that the 3-week placement was short-term and insufficient to experience and practice rural dentistry
Branson BG, et a.l; 2007/ USA [38]	University of Missouri-Kansas City	Original research	Dental hygiene student rotations to rural and underserved areas	Students’ competencies and satisfaction/Qualitative and quantitative (questionnaire) measurements	Increased satisfaction among students Improved students’ clinical competencies
Hunter ML, et al.; 2007/ UK [52]	Wales College of Medicine	Original research (pilot study)	Community dental service outreach teaching program for final year dental students for providing pediatric dental care	Students’ confidence in comparison to the clinical session at dental school/Pre- and post-questionnaires	Students showed higher confidence in providing comprehensive oral care
Lo ECM, et al; 2007/ China [81]	University of Hong Kong	Original research	3-year outreach dental service program in four primary schools in rural town in southern China (partnered with WHO Collaboration Centre on Primary Health Care)	Cost-effectiveness/ Descriptive measurement	Cost-effective and affordable
Macnab J, et al.; 2008/ Canada [62]	University of British Columbia	Original research	Same as Harrison RL, et al.; 2006 [61], three years’ evaluation	Improvement in oral health and oral health knowledge among school children/Oral examination (oral health indices)	Reduced DMFT/dmft score and more caries-free children after three years Dentist noticed improved oral health and knowledge among children
Schoo AM, et al; 2008/ Australia [33]	Flinders University and Deaking University	Original research (pilot study)	Medical, dental, nursing and allied health students were enrolled in the rural placement program	Rural recruitment of new graduates/Pre- and post-survey questionnaires	Positive association of the rural placement with rural recruitment More urban students began rural practice compared to rural
Shrestha A, et al.; 2008/ India [75]	Manipal College of Dental Sciences	Original research (cross-sectional survey)	Weekly and monthly conduction of rural outreach dental camps	Patient satisfaction after one year/Questionnaire	High patient satisfaction
Abuzar MA, et al.; 2009/ Australia [25]	University of Melbourne	Original research	Rural dental rotation program for education and training of final year dental students	Dental students’ experience/Anonymous questionnaires	Students had a positive perception, enriched feeling towards community’s culture and better understanding of community’s dental needs
**Author; Year/ Country**	**University/ Institution**	**Type of publications**	**Program description**	**Outcome variable/ Measurement instrument**	**Results**
Andersen RM, et a.l; 2009/ USA [55] Friedman JA, et al.; 2009/ USA [56] Kuthy RA, et al.; 2009/USA [58] Thind A, et al.; 2009/ USA [57]	National Pipeline schools (11): Universities of Boston, Howard, Temple, Ohio State, South Carolina, Connecticut Health Center, Washington, West Virginia, University of California at San Francisco, University of Illinois at Chicago, and Meharry Medical College California Pipeline schools (4): Universities of Pacific, Southern California, University of California at Los Angeles, and Loma Linda University	Original project	USA Dental Pipeline Project– 2001 to 2010, one of the most extensive projects that involved various dental schools aimed to increase the recruitment and retention of students from under-represented minorities (URM) and low-income groups in dental schools	Impact on URM enrollment; developing community-based dental education curricula; and extending extramural clinical rotations/ Descriptive measurement	Increased enrollment of URM students by 27%, URM students planned to include at least 25% of minority groups as their patients, more diverse dental students’ recruitment, provided context for developing more culturally competent providers URM students noted difficulties such as financial barriers, high education debts, location of dental schools far from their homes, high cost of urban living, perceptions that their dental school was unwelcome, and that schools lacked URM role models Increased extramural facilities and students’ rotations Curricular changes included community-based dental education courses with considerable changes in content, teaching and evaluation methods
Fricton J, et al.; 2009/ USA [84]	University of Minnesota	Original project (as a chapter in Dental Clinics of North America)	University of Minnesota Tele-dentistry Project using real-time video conferencing	Acceptance and satisfaction among patients and providers/Questionnaire	Increased acceptance and satisfaction among patients and providers
Skinner JC, et al; 2009/ Australia [54]	Charles Sturt University	Original research report	Charles Sturt University Dentistry program for rural students to study and practice dentistry in rural areas	Rural recruitment and retention of graduates/ Descriptive measurement	The project estimated up to 60% retention of the first cohort of 2014 graduates in the rural areas
Arevalo O, et al.; 2010/ USA [91]	University of Kentucky	Original research (cost analysis)	Dental Outreach Programs Kentucky: four mobile dental clinics for elementary school children and Head Start children in several rural counties	Financial feasibility of mobile dental units/ Financial analysis	Financially, mobile units were good option for accessing rural underserved population Ongoing program Conducted many successful outreach activities with improved oral health of children
McFarland KK, et al.; 2010/ USA [49]	University of Nebraska	Original research (retrospective study)	Analysis of dental students’ attitudes from 1989 to 2008 about rural practice	Pattern of dental students working in rural practice after graduation/ Descriptive measurement	More non-residents than residents and more women than men, who remained in the state after graduation, were located to practices in rural communities

**Table 5:** Summary of published research articles identified in the scoping review (2011 onward).

**Author; Year/ Country**	**University/ Institution**	**Type of publications**	**Program description**	**Outcome variable/ Measurement instrument**	**Results**
Bhayat A, et al.; 2011/ South Africa [67]	University of Witwatersrand	Original research project report	Final year dental students were enrolled in two groups for outreach: Phelophepa train, a mobile primary health care Public oral health facility	Dental students’ competencies/ Mixed method (qualitative and quantitative questionnaires)	Improved clinical skills and efficiency and understanding of the community needs Problems: High patient input and long working hours on the train, and inadequate and non-functioning equipment in public oral health facility
Summerfelt FF; 2011/ USA [64]	University of Northern Arizona	Original research	Pilot teledentistry program having dental hygiene students as mid-level practitioners in rural areas	Pedodontist’s acceptance during initial field trial/Opinion Evaluation of diagnostic efficacy of patients’ digital X-rays taken by dental hygiene students at two remote locations/ Descriptive measurement Faculty and students’ opinion/Survey questionnaires	Pedodontist identified project as successful No significant difference between diagnostic efficacy at two remote sites Hygiene students rated digital X-ray training as ‘excellent’ Hygiene students and faculty members rated value of inclusion of teledentistry skills into dental hygiene curriculum as ‘excellent’
Martinez-Mier, et al.; 2011/ USA [43]	Indiana University	Original research	Hidalgo International Service-learning programme with multidisciplinary students and faculty- dental, medical, nursing, public health and social work	Students’ and faculty opinion about program development/ Questionnaires and SWOT analysis	Students acquired better clinical skills and appreciated rural culture and barriers to access to care Faculty reported better understanding of students’ learning and better communication skills among team members
Parlani S, et al; 2011/ India [71]	Chhatrapati Sahuji Maharaj Medical University	Original research	Awareness programs of prosthodontics among aging rural population	Evaluate awareness among study population/Interview and quantitative assessment	Increased awareness of ageing population
Bulgarelli AF, et al.; 2012/ Brazil [50]	University of São Paulo	Original Research	Huka-Katu (beautiful smile) culturally adapted outreach programs in an Indigenous community for final year dental students	Dental students’ competencies and experiences/Qualitative interview	Improved students’ understanding of primary oral health care Students developed sense of cultural respect and social perspective
Glassman P, et al.; 2012/ USA [85]	University of Pacific	Original research (First phase of demonstration project)	Virtual Dental Home program (Expansion of dental home concept with use of advanced telehealth technology by teamwork between registered dental auxiliaries and distant dentists	Impact of implementation of project’s first phase/Descriptive analysis and feedback	750 patients received preventive and early intervention dental treatments Staff, caregiver, and parent education led to increased dental literacy and compliance with daily oral health practices, treatments, and referrals
**Author; Year/ Country**	**University/ Institution**	**Type of publications**	**Program description**	**Outcome variable/ Measurement instrument**	**Results**
Macnab A, et al., 2012/ Uganda [72]	Joint Ugandan/ Canadian university (University of British Columbia and Makerere University)	Original research intervention study (project report)	‘Many voices, one song’: oral health model of health promoting schools based on Brighter Smiles program for the Aboriginal community in Canada. It included health education by local teachers and the university team and daily in-school tooth brushing	Change in oral health knowledge and oral health status after four years’ evaluation/Qualitative and quantitative methods (questionnaire and interviews of teachers)	Improved oral health and health-related knowledge among children Positively influenced university faculty and students
Parker EJ, et al.; 2012/ Australia [63]	University of Adelaide	Original research evaluation study	Aboriginal children’s Dental Program in Port Augusta by dental therapists and dentists; Integrated health project involving health promotion by conducting a workshop for Aboriginal health workers by dental students with key role of local primary health care provider (in collaboration with Pika Wiya Aboriginal Health Service)	Change in participation rate after 3.5 years and identified challenges/Documented data and interviews	Increased rate of participation for dental care for Aboriginal children from 53% to 70%. Main challenges: Difficult to contact patients, communicate with parents or guardians, missed appointments, and consent-related issues
Tandon S, et al.; 2012/ India [73]	Manipal College of Dental Sciences	Original research	Mobile dental health care services in rural areas	Knowledge, attitude, practice, and satisfaction among rural people after three months/Questionnaire	Improved oral health knowledge, attitude, and practices High patient satisfaction
Vashisth S, et al.; 2012/ India [77]	Swami Devi Dayal Dental College	Original research (retrospective study)	Various outreach programs in rural areas	Type of patients, diseases, and services at outreach for three months/Descriptive measurement	Dental caries was prevalent mostly curative services were provided recommended development of need-based programs
Johnson G, et al.; 2011/ Australia [27]	University of Sydney	Original research	1-month duration of Rural Placement Program was initiated for 4^th^-year dental students.	Students’ competencies and experience/Pre-and post-questionnaires	Positive rural experience Improved clinical skills Increased chances for considering rural practice after graduation
Johnson G, et al.; 2012/ Australia [1]				Competencies of placement students compared to non-placement students/Pre-and post-questionnaires	Positive attitude and improved clinical skills compared to non-placement group
Johnson G, et al.; 2013/ Australia [28]				Staff and supervisors’ attitude/Interviews	Supervisors recognized students’ positive clinical and personal development and identified the program as feasible
Johnson G, et al.; 2013/ Australia [29]				Follow up for rural recruitment after three years/Descriptive measurement	Higher proportion of graduates worked in rural location compared to non-placement group
**Author; Year/ Country**	**University/ Institution**	**Type of publications**	**Program description**	**Outcome variable/ Measurement instrument**	**Results**
Dawkins E, et al.; 2013/ USA [86]	University of Western Kentucky	Original research	Free dental sealant and oral examination program through mobile dental unit for school children since 2001[86].	Compare sociodemographic characteristics between caries and non-caries group and explore factors responsible for non-treated caries in children from 2006-2011/Descriptive measurement	More non-treated caries were observed in children living in rural areas, without private insurance and having older ages
Ibiyemi O, et al.; 2013/ Nigeria [40]	University of Ibadan	Original research project report	Ibarapa Community Oral Health Programme: 6-week rural posting program for fifth-year dental students at Igboora	Dental students’ attitude/Reports	Students’ expectations from the program were fulfilled Students became sensitized to community needs Enhanced teamwork skills
Lalloo R, et al.; 2013/ Australia [30]	Griffith University	Original Research	Remote rural clinical placement in Indigenous Community over three years from 2009 to 2011	Audited reports of services provided/Descriptive measurement	Primarily offered clinical examination, restorative, and oral surgical services and provided fewer preventive and periodontal services
Lalloo R, et al; 2013/ Australia [31]				Students’ perception and competencies/Online questionnaire survey	Students had positive experience, improved clinical competencies, gained knowledge and developed cultural sensitivity
Lalloo R, et al.; 2013/ Australia [32]				Auditing of expenditure/ Cost analysis	Factors related to financial support overshadowed benefits to students and local community, e.g. additional cost for salary incentives, travel, accommodation and meals
Chandrashekar B, et al.; 2014/ India [70]	Kamineni Institute of Dental Sciences	Original research (intervention study)	Oral health promotion intervention study for six months with children divided into four groups: 1: Control group: no subsequent education 2: Education by a qualified dentist at every three months 3: Education by the trained school teachers with oral hygiene screening 4: Intervention 3 + children were given the oral hygiene aids	Pre- and post-oral health status using oral health indices/Descriptive measurement	Improvement in children’s’ oral hygiene status in oral hygiene aids group Regular dental education sessions by school teachers were more efficient compared to an occasional meeting by dentists
Goel P, et al.; 2014/ India [92]	Rajasthan Dental College	Original research	Indigenously fabricated mobile portable dental unit	Measurement of cost efficiency after seven years’ evaluation/ Descriptive method	Cost-effective, easy to transport and feasible Required additional space and time for set up
**Author; Year/ Country**	**University/ Institution**	**Type of publications**	**Program description**	**Outcome variable/ Measurement instrument**	**Results**
Naidu A, et al.; 2014/ Canada [80]	McGill University	Original research	Community-based participatory research to promote oral health of school children in a rural Aboriginal community	Explore oral health practices and development of oral health promotion activities/Semi-structured interviews	Successfully developed culturally appropriate methods for oral health promotion by engaging children with their parents
Nayar P, et al.; 2014/ US [46]	University of Nebraska	Original research	Rural community- based dental education program for dental students to improve their competencies.	Attitude of supervising dentists regarding program effectiveness for improving student’s competencies/ Electronic survey questionnaire	Enhanced dental students’ skills while experiencing the real-world situations Supervising dentists considered program as successful and rated it as ‘excellent’ or ‘very good’
Anderson VR, et al.; 2015/ New Zealand [34]	University of Otago, New Zealand	Original research report	Oranga Niho dental student outplacement project for final year dental students	Attitude of students, supervisors and clients and their caregivers/Mixed method (quantitative by pre- and post-questionnaires for dental students and qualitative by paper questionnaire for adult’s clients and caregivers)	Students showed readiness for the outplacement and willingness to work for Maori communities Majority of supervisors expressed students’ readiness for working in remote areas Most patients and their caregivers had positive attitudes about students’ care
Asawa K, et al.; 2015/ India [87]	Pacific Dental College and Hospital	Original research (retrospective study)	Dental outreach programs for rural population through mobile dental units	Number of patients, diseases, services in outreach as well as the effectiveness of referral from 2 years’ data/Descriptive measurement	Dental caries, periodontal disease, and dental fluorosis were prevalent Generally curative services were provided Increased effectiveness of referral system
Okeigbemen SA, et al.; 2015/ Nigeria [78]	University of Benin	Original research (retrospective study)	Rural outreach dental clinic	Dental service utilization and trends of patients attending this program/Descriptive measurement	Lower utilization of dental services Recommended the need for oral health promotion and preventive services through frequent outreach activities
Vashishtha V, et al.; 2015/ India [76]	D.J. College of Dental Sciences and Research	Original research (cross-sectional study)	Community dental outreach programs for the 1-month duration	Patient satisfaction/ Questionnaire	High patient satisfaction
Abuzar MA, et al.; 2016/ Australia [26]	University of Melbourne	Original research (case study)	Aboriginal community oral health placement for final year DDS and BOH (Bachelor of Oral Health)	Students’ experience towards program from 2008-2014/Questionnaire survey	Students valued Aboriginal culture Increased chances for recruitment Students appreciated clinical supervisors and staff
**Author; Year/ Country**	**University/ Institution**	**Type of publications**	**Program description**	**Outcome variable/ Measurement instrument**	**Results**
Okeigbemen SA; 2016/ Nigeria [48]	University of Benin	Case study	Clinic-based curriculum for the dental students	Effect of community- based services on rural dental services/Quantitative and qualitative methods	Improvement in community-based dental services for rural residents such as awareness, screening, and referral services.
Shannon CK, et al.; 2016/USA [36]	University of West Virginia	Original research (survey)	6-week community-based rotations for senior dental students from 2001–2012	Students’ assessment of predictors for practicing in rural areas and intention for rural recruitment/Online pre- and post-survey questionnaires	Students identified significant predictors before rotations: expectations for rural practice, rural hometown, and more practice accessibility to poor patients Increased likelihood of rural practice after rural rotations
Verma A, et al.; 2016/ India [39]	M.R. Ambedkar Dental College, V.S. Dental College, and M.S. Ramaiah Dental College	Original research (Non-randomized trial)	Outreach program where dental interns were divided into outreach group and dental school-based group	Pre-and post-evaluation of students’ confidence and communication skills/Questionnaire	Higher confidence and communication skills among outreach group students

**Table 6:** Summary of non-research publications including relevant web records identified in the scoping review.

Author; year/ Country	University/ Institution	Program description	Outcome variable/ Measurement instrument
The S-Miles To Go Mobile Dental Program; 1997/ USA [90]	Buffalo University	Mobile dentistry program for rural Chautauqua County children	Program has been successful in meeting this population’s oral health needs
Dental Training Expanding Rural Placements (DTERP) Program; 2013/ Australia [24]	Universities of Adelaide, Melbourne, Sydney, Western Australia, Queensland, Griffith University, Flinders University	Program to improve rural access to dental services by expanding dental training through placements in rural settings	Outcome variables: 6-monthly performance and expenditure report, financial statements yearly and at the end of the project, final performance report at the end of the project Expected an increase in rural dental workforce after students’ regional or rural clinical training No results
NHS Education for Scotland; 2014/ Scotland [53]	Scottish Universities, e.g. University of Dundee, University of Edinburgh, and University of Glasgow	Scottish Dental Postgraduate Training Fellowship program, an initiative for rural Scotland	Expected to help intermediate and higher-level care in rural areas of Scotland by providing dental surgeons in these areas No results
RIDE: UWSOD Regional Initiatives in Dental Education; 2015/ USA [45]	University of Washington	Regional Initiatives Project in Dental Education for improving oral health access by increasing number of dentists	Ongoing program increased access to dental care to this rural population Exposed students to real-world experiences
Better Oral Health in European Platform; 2015/ Malta [66]	University of Malta	‘Our Drive for a Healthy Smile’ with the help of a mobile dental clinic	Helped in reducing oral health inequalities and providing dental students an opportunity to understand the community needs
European Commission; 2016/ Romania [89]	SAN-CAR—mobile dental health care with Constanta’s Ovidius University	SAN-CAR—mobile dental health care for rural communities in Romania and Bulgaria	Improved oral health and quality of life of rural population
Poche Centre for Indigenous Health- 5 Year strategy, Strategic plan 2016–2020: on Healthy Kids, Healthy Teeth, Healthy Hearts; 2016/ Australia [83]	University of Sydney	Healthy Kids, Healthy Teeth, Healthy Hearts program: To improve health services and capacity- and skill- building	Helped in establishing dental services by working with existing Aboriginal Health Services Created employment opportunities by engaging local Aboriginal people to deliver oral health services

#### Measuring instruments for outcomes

Three main approaches have been used to evaluate the programs: quantitative, qualitative and mixed. In the quantitative approach, instruments such as questionnaires (closed and open-ended, pre- and post-, anonymous, electronic online) [1, 3, 11, 25–27, 31, 33, 35, 36, 39, 46, 51, 52, 60, 64, 65, 73, 75, 76, 82, 84, 85], oral examinations [61, 62, 74], health and oral health-related indices [69], descriptive measurements [29, 30, 40, 43, 49, 54–58, 64, 65, 68, 70, 74, 77, 78, 81, 85–88], measurement of grades [37] and SWOT (strength, weakness, opportunities and threat) analyses [43] were used. Additionally, quantitative measurements, such as cost per patient, marginal cost and cost analysis [32, 41, 42, 79, 91, 92] were used to measure the cost-effectiveness of various programs. For qualitative measurement, tools, such as data documentation [63], interviews [28, 47, 50, 63, 80] and mixed approaches, questionnaires in combination with focus group discussions, interviews and open-ended questionnaires [34, 38, 48, 59, 67, 71, 72] were used to measure the outcomes.

#### Outcome variables

1. **Outcome variables for training and education programs**
  These included students’ competencies and experience [1, 3, 11, 25–27, 31, 34, 38–43, 47, 50–52, 60, 64, 65, 67], supervising dentists’ and students’ satisfaction [35, 38, 84], staff and supervisors’ attitude, experience, and feasibility [3, 28, 34, 43, 46, 64, 85], client/patient and caregivers’ attitude [34], attitudes of health workers [59] and student evaluations by supervising dentists [37]. Also, several impacts were observed, such as effects on students’ education, research and oral health services [44], impact on rural recruitment and graduate retention [24, 29, 33, 36, 49, 54] and on minority and rural student enrollment [55–58].
2. **Outcome variables for oral health service related programs**
  These outcome variables included community acceptance [82], identification of challenges [63], knowledge, attitude and satisfaction among patients [72, 73, 75, 76], changes in oral health practices [80], changes in oral health status [41, 42, 61, 62, 70, 72, 74, 86], effect on oral health services [48, 77, 87, 88] and utilization of services [63, 68, 71, 74, 78].
3. **Other outcomes**
  These variables consisted of audited reports of services provided [30], cost-effectiveness [41, 42, 79, 81, 91, 92] and expenditures [32, 74].

### Program evaluation results (Tables 3–6)

Outcomes of rural oral health initiatives and their impact varied among these programs. Accordingly, most of the training and education programs were shown to be feasible through feedback from staff, academic personnel, and trainees. For example, these programs were reported to have helped improve students’ and trainees’ clinical competencies and social sensitization, and provided them with positive experiences and satisfaction [1, 3, 11, 25–27, 31, 34, 35, 37–43, 47, 50–52, 59, 60, 64, 65, 67, 84]. Staff and supervisors noted positive attitudes and experiences, as well as satisfaction with and feasibility of these programs [3, 28, 34, 35, 38, 43, 46, 64, 84, 85]. Also, the programs demonstrated an increased enrollment, recruitment and retention of dental students in rural and remote areas [24, 29, 33, 36, 49, 54–58] and cost-effectiveness [41, 79, 81, 91, 92]. The clients/patients and caregivers of these training programs had experienced positive attitudes and acceptance of these initiatives [34]. Furthermore, oral health service-related programs had identified and reported community acceptance [82], improved knowledge, attitude and satisfaction among patients [72, 73, 75, 76], improved oral health practices [80], better oral health status [41, 42, 61, 62, 70, 72, 74, 86], improved quality of oral health services [48, 77, 87, 88] and enhanced utilization of services [63, 68, 71, 74, 78].

These oral health care services included the provision of more interventional procedures compared to preventive and improved referral services. A few programs reported barriers to these outcomes, such as short duration, deeming them insufficient to experience and practice rural dentistry [3].

## Discussion

In most of the countries, rural-urban health disparities are seen not only in dentistry but also in other health disciplines namely medicine, pharmacy, nursing. It is mostly linked to the disproportionate distribution of health care providers including dentists, physicians, nurses, and pharmacists [8, 93]. Government organizations, for-profit and non-profit non-governmental organizations and academic institutions around the world have taken several steps towards improving access to rural dental care. In this extensive literature scoping review, we have reported evidence of academic institutes’ initiatives in improving access to oral health care for rural and remote communities.

Outcomes of this scoping review revealed that students benefitted from these university initiatives by having opportunities to work in real-world situations that inspired them to learn [46], practice various procedures, manage the diversity of patients and gain experience working in a team [26]. Indicators for the success of these programs were: students’ satisfaction with the program, community-based experience, enhanced communication skills and self-confidence; a high rate of treated patients; reduced oral health problems in rural areas after rural placements; and an increased percentage of students working in rural dental practices [1, 3, 11, 24–27, 29, 31, 33–43, 47, 49–52, 54–65, 67, 68, 70–72, 74, 78, 84, 86]. The effectiveness of rural exposure through training in universities and institutions was found to vary due to reasons such as the short duration of rural placement programs, as well as a lack of standardized methodologic and evaluation tools [94]. According to Lalloo et al., confidence among dental students in choosing a dental practice in rural areas was the most relevant outcome measure of the impact of students’ rural placement programs [31]. Orpin et al. commented that the subsequent fair distribution of the rural workforce would be the ultimate test in evaluating the effectiveness of these kinds of programs, although that would be a long-term vision [94]. Rural areas, by virtue of being smaller, offer better opportunities for any program to be successful due to logistical ease of administrative coordination and collaboration, with less organizational and managerial impediments than in urban settings [16].

Most of the mobile dental clinics, dental camps, and dental outreach programs successfully disseminated awareness, provided treatment and enhanced access to care for people living in rural areas. Results from the various outreach programs showed that they could assist in bridging the wide gap created between rural residents’ actual dental needs and their demand for dental care [71, 73, 75–78, 87]. Integration of telehealth into rural oral health services is likely to be successful, but more time is needed to realize the full oral health implications of rural E-health technology [16].

In most of the programs, universities received funding from various sources, but some programs could not be continued due to lack of funding [63, 68]. If the necessary funds become available, it is expected that these services could be provided at a marginal cost when compared to the costs of similar treatments provided by either public-sector staff or private practitioners [79]. The strong motivation of academia’s initiatives to improve oral health care access for rural and remote communities appears to be justified by their positive and effective results; however, long-term evaluations by the institutes and their partners are crucially needed. Most often, curative services were provided in these programs; hence, there is a need to shift our focus towards preventive and promotional oral health services to achieve the global vision of eliminating oral health disparities among rural and remote communities.

Training undergraduate dental students has the potential to improve dental services in rural areas, particularly in areas with limited or no publically-funded dental services [79]. The total cost of the services provided by students, including their travel, living and supervision, is lower than that of private dental providers [79]. The results of our scoping review suggested that very few outreach programs were found to be cost-effective [41, 81, 92]. These programs not only significantly reduced the cost of setting up dental clinics or mobile dental clinics but also further lower costs by using available local resources and staff, such as school teachers [81]. However, long term evaluation are required to determine true cost-effectiveness of these programs. One study demonstrated the cost-effectiveness of a rural outreach program using a portable dental unit [92]. The cost of dental services provided by students with mobile dental units may be high initially, but they become cost-effective over time. [41].

The types of academic initiative programs stated in our scoping review benefited both the rural communities and the academic institutions. Rural residents gained access to dental services and students from the academic institutions gained experience in their field and had an opportunity to develop clinical practice skills by providing care to a broad range of patients.

The WHO has provided strategies and recommendations on improving access to health workers in rural and remote areas [18]. According to these strategies, medical and dental schools were identified as playing a major role by enrolling students from rural backgrounds and establishing professional schools in rural areas or on the outskirts of major cities [18]. WHO also recommended students’ clinical rotations in rural areas, as well as introducing rural health issues in the curriculum [18]. Among these WHO recommendations [18], results from our scoping review reveal the major contribution of such institutions through student rural rotations and by enrolling students from rural areas for health promotion activities, thereby reducing cost and related expenditures. However, some countries like Australia has established new dental schools predominantly in rural and remote areas with the aim of increasing the recruitment of rural students, and ultimately providing a rural workforce.

Our scoping review identified the following gaps in the existing literature on academic initiatives in rural and remote areas. These include great variability in program design, duration, data collection tools (often non-standardized), more focus on curative dental services as opposed to preventive or promotive services and lack of sustainable financial support.

### Limitations

The main limitations of this scoping review are twofold. Firstly, the literature review was restricted to articles written in English only. There is likely published work in some other areas of the world like Europe and South America in other languages. Secondly, these publications were not assessed specifically for scientific quality; thus, the results of this scoping review should be interpreted carefully.

### Recommendations

These findings point to the following empowering ‘next steps’ for international universities and training institutes: development of international partners to conduct long-term program evaluations; create a mandate to expand and sustain rural residency programs; build strong partnerships with public and private health sectors; promote interdisciplinarity of rural health provision; and build links with policy makers to mobilise the support, development and implementation of universal academic rural and remote oral health programs. Future programs could be customized to address the disparities for a country’s or region’s rural health care needs while considering the administrative, educational and fiscal structure of dental faculties and their universities.

## Conclusion

This scoping review describes university-based initiatives in improving access to oral health care in rural and remote regions. The results suggest that these innovative programs are transferable and may serve as valuable models for other academic institutions to promote the oral health of rural and remote populations and improve their right of access to oral health care.
